# Clever generation of rich SPARQL queries from annotated relational schema: application to Semantic Web Service creation for biological databases

**DOI:** 10.1186/1471-2105-14-126

**Published:** 2013-04-15

**Authors:** Julien Wollbrett, Pierre Larmande, Frédéric de Lamotte, Manuel Ruiz

**Affiliations:** 1 CIRAD, UMR AGAP, Montpellier F-34398, France; 2 IRD, UMR DIADE, Montpellier, France; 3 INRA, UMR AGAP, Montpellier F-34398, France; 4 Institut de Biologie Computationnelle, 95 rue de la Galéra, 34095, Montpellier, France

## Abstract

**Background:**

In recent years, a large amount of “-omics” data have been produced. However, these data are stored in many different species-specific databases that are managed by different institutes and laboratories. Biologists often need to find and assemble data from disparate sources to perform certain analyses. Searching for these data and assembling them is a time-consuming task. The Semantic Web helps to facilitate interoperability across databases. A common approach involves the development of wrapper systems that map a relational database schema onto existing domain ontologies. However, few attempts have been made to automate the creation of such wrappers.

**Results:**

We developed a framework, named BioSemantic, for the creation of Semantic Web Services that are applicable to relational biological databases. This framework makes use of both Semantic Web and Web Services technologies and can be divided into two main parts: *(i)* the generation and semi-automatic annotation of an RDF view; and *(ii)* the automatic generation of SPARQL queries and their integration into Semantic Web Services backbones. We have used our framework to integrate genomic data from different plant databases.

**Conclusions:**

BioSemantic is a framework that was designed to speed integration of relational databases. We present how it can be used to speed the development of Semantic Web Services for existing relational biological databases. Currently, it creates and annotates RDF views that enable the automatic generation of SPARQL queries. Web Services are also created and deployed automatically, and the semantic annotations of our Web Services are added automatically using SAWSDL attributes. BioSemantic is downloadable at http://southgreen.cirad.fr/?q=content/Biosemantic.

## Background

Currently, the large amount of plant high-throughput data that have been produced by different laboratories is distributed across many different crop-specific databases. Plant biologists and breeders often need to access several databases to perform tasks such as locating allelic variants for genetic markers in different crop populations and in a given environment or investigating the consequences of a mutation at the transcriptome, proteome, metabolome and phenome levels. The integration of these disparate databases would make complex analyses easier and could also reveal hidden knowledge [1,2].

However, biological data integration faces challenges because of syntactic and semantic heterogeneity. In their reviews, Stein LD [3] and Goble C & Stevens R [4] provide a fair criticism of the lack of integrated approaches and provide a similar vision for the future, which is that the Semantic Web (SW) can aid in data integration. According to the W3C, “the SW provides a common framework that allows data to be shared and reused across applications, enterprises, and community boundaries”^a^. The SW currently provides recommendations (RDF [5], SPARQL [6], OWL [7]) for enabling interoperability across databases. Furthermore, major plant databases, such as TAIR [8], Gramene [9], IRIS [10], MaizeGDB [11] and GnpIS [12], annotate their data using ontology terms to link different datasets and to facilitate queries across multiple databases. Guided by life science integration studies [13,14], annotating data with ontologies promotes the development of ontology-driven integration platforms [15,16].

In parallel, Web Services (WS) are becoming an increasingly popular way of establishing robust remote access to major bioinformatics resources, such as EMBL-EBI, KEGG and NCBI. WS are virtually platform-independent and are easily reusable. Indeed, analysis and data retrieval WSs can be rapidly combined and integrated into complex workflows.

The common use of the SW and WS standards has the promise of achieving integration and interoperability among the currently disparate bioinformatics resources on the Web [17]. There are currently existing efforts to describe Web Services with semantic annotations by using ontologies, such as SSWAP [18], SADI [19] and BioMoby [20]. However, none of these approaches are focused on the automation of business logic [21]. The implementation of new Semantic Web Services (SWS) can be time-consuming and requires the developer to know how to manipulate SW and WS standards and to have expertise on the database schema. To our knowledge, there are currently no ongoing efforts in the context of the automation of SWS creation that are both specific to relational databases and based only on W3C standards.

Our goal is to develop a framework for the creation of SWS for the field of biology by using both SW and WS technologies.

Bio-ontologies result from community reflexions in which each term and each relation are explicitly defined for an application domain. Biological data are annotated with terms from these ontologies, which add a semantic component to them. In BioSemantic, semantics is given by annotation with ontological terms of heterogeneous relational databases schema. These annotations will be used for automatic SWS creation. They will also be used to add semantics to these SWS by annotating their interfaces (input and output).

To make the process of WS development as easy as possible, we have developed a semi-automated framework to accelerate the development of SPARQL queries for relational databases. These queries are automatically added to SWS backbones allowing an easier integration of distributed relational databases. This article focuses on biological relational databases, but because of using only SW and WS standards, BioSemantic can potentially be applied to other science fields.

## System and methods

### BioSemantic framework overview

**Figure 1:**
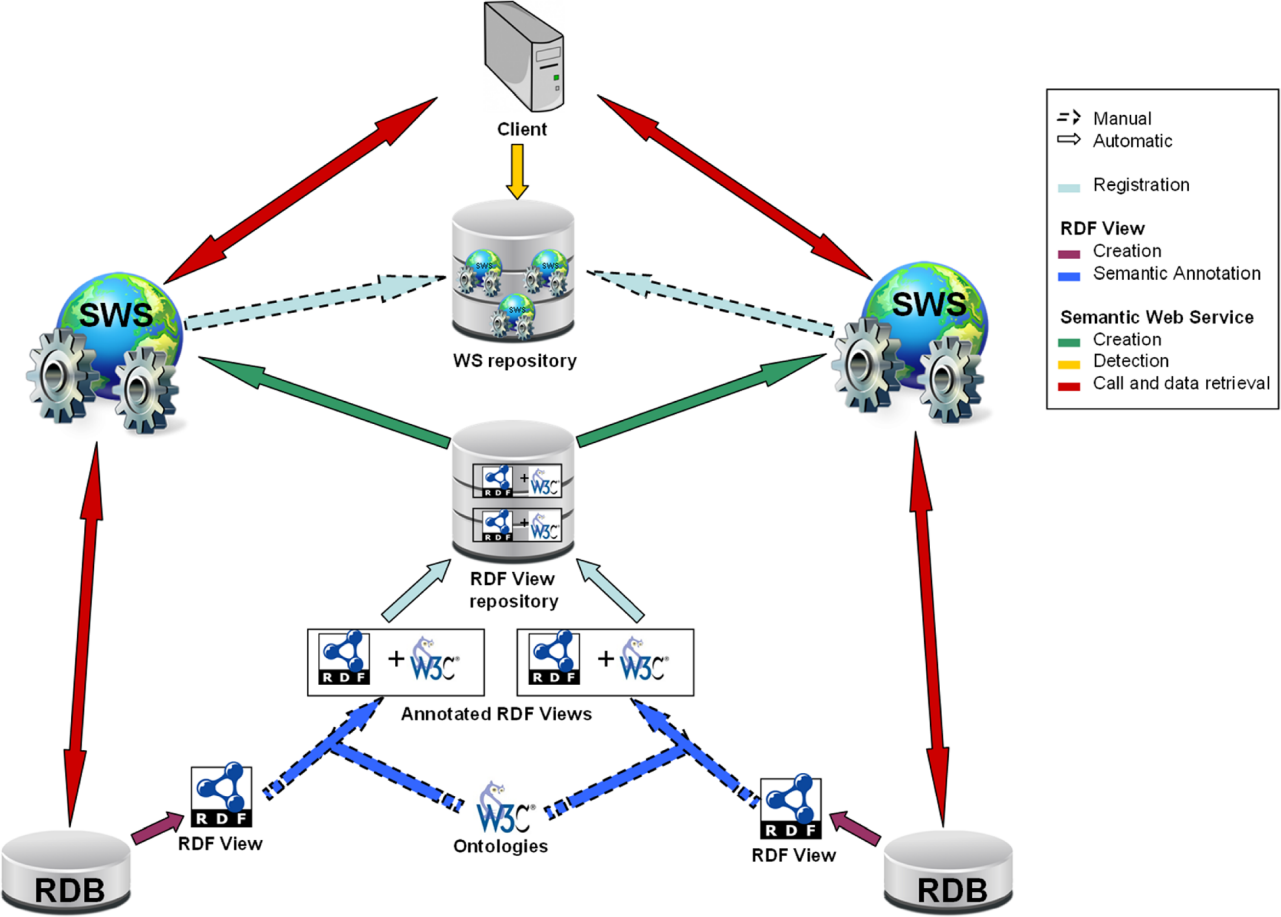
**Global architecture of the BioSemantic framework.** The first contribution of our work is the automatic creation of an RDF view containing RDF metadata, which is necessary for the automatic creation of Semantic Web Services. The second contribution is the automatic creation and deployment of Semantic Web Services.

The overall architecture of the BioSemantic framework is shown in Figure 1. One advantage of this architecture is that its decoupling takes place in two different steps, which might be achieved by different user profiles. In the first step, the data provider must publish the schema of its relational database. First, the local RDF view of the database schema is automatically created for each relational database to be integrated. Then, the RDF view must be manually annotated by experts with terms from existing bio-ontologies. The RDF views, both created and annotated, are stored in an RDF repository. Once the RDF view is available, the second step is the creation of the SWS. This step is uncoupled from the first step and could be realised by a data consumer without any knowledge of the database schema. The previous semantic annotations of RDF views are used to automatically create SWS containing SPARQL queries and to use the bio-ontological terms as input/output. SWS are then stored in a Semantic Web Services repository, from which they can be easily detected by clients. These clients can use the SWS as wrappers to overstep the heterogeneity of the relational databases.

**Figure 2:**
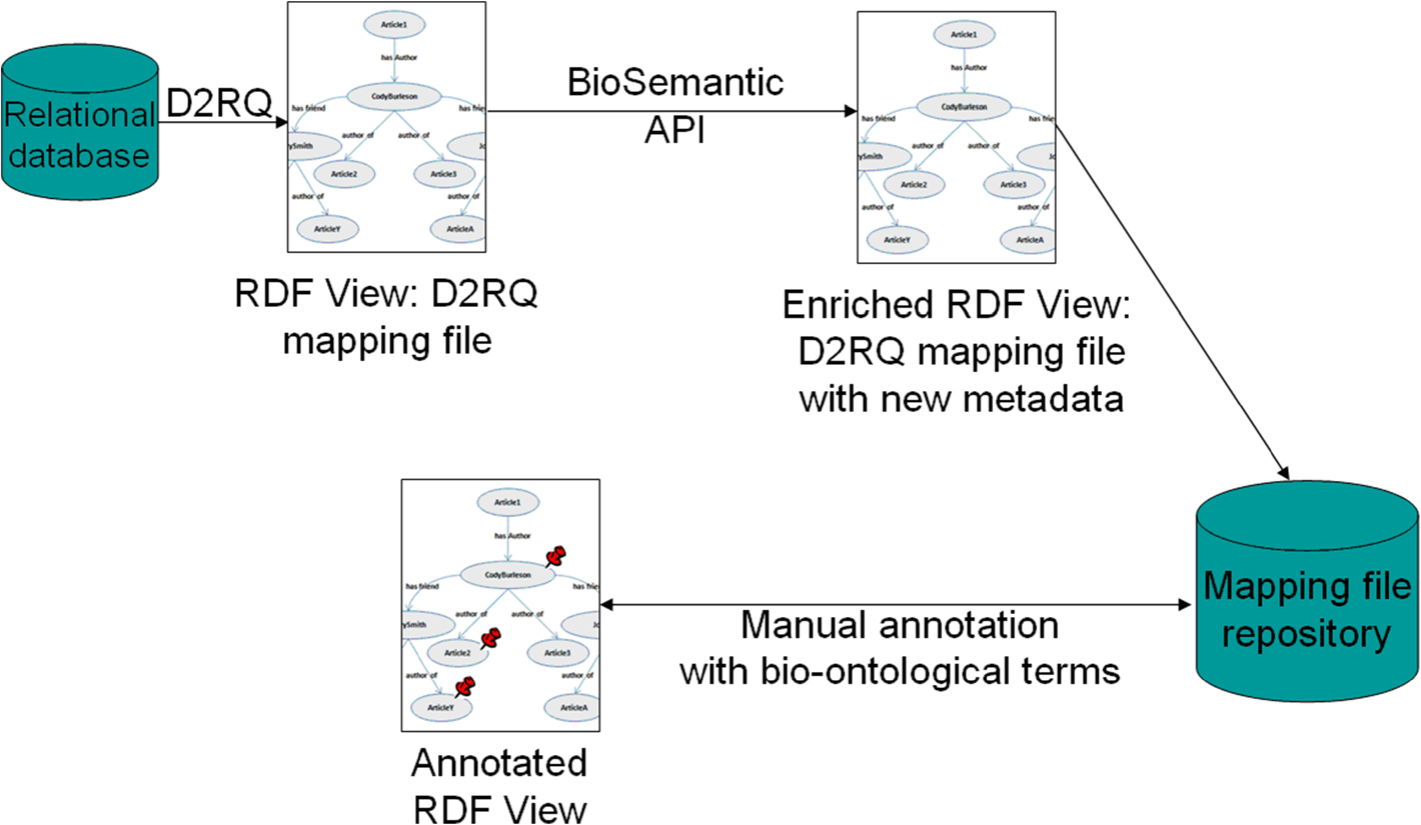
**Generation and semi-automatic annotation of the RDF view.** D2RQ creates the D2RQ mapping file, and our BioSemantic API automatically adds new metadata about the database schema. Finally, the mapping file is stored in a repository. Annotation with bio-ontological terms is performed manually by an expert.

**Figure 3:**
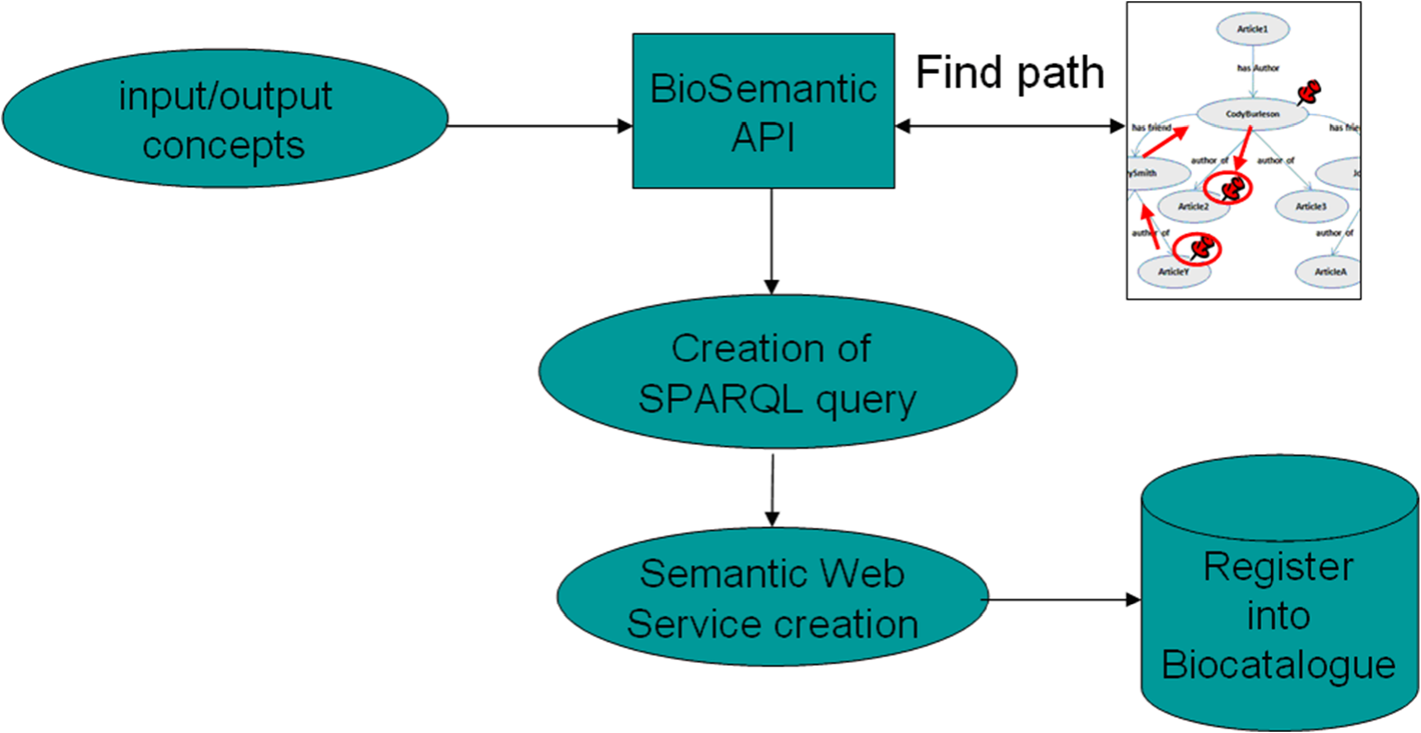
**Automatic generation of Semantic Web Services.** The Web Services developer selects the bio-ontological terms to be used as input/output. All of the mapping files, which are stored in the mapping file repository, are automatically parsed to find a path linking the input and output ontological terms. If such a path is found, it is used to create a SPARQL query. The query is integrated into a semantic Web Service that is then registered in a Web Service registry, such as BioCatalogue.

We will detail below the entire process for generating a BioSemantic SWS, which can be divided into two main parts: *(i)* the generation and semi-automatic annotation of an RDF view (Figure 2) and *(ii)* the automatic generation of the SWS (Figure 3).

### Generation and semi-automatic annotation of an RDF view

#### Relational database-to-RDF mapping

The research in the domain of mapping between databases and ontologies is very active and corresponds to various motivations and approaches [22]. In BioSemantic, we use the mapping as an intermediate layer between the user and the stored data. This layer provides an abstraction of the database and allows the user to query databases without knowledge of the database schema. These characteristics correspond to the motivation known as “data access based on ontology”. For that purpose, we found only two tools that strictly use SW standards: Virtuoso [23] and D2RQ [24-26]. We have chosen D2RQ because this tool is open source, easy to use and all of the needed functionalities are free. In addition, some bioinformatics projects have successfully used D2RQ. With D2RQ, we can automatically generate a mapping file that provides an RDF view of the database schema.

#### RDF view description

The RDF view created by D2RQ can be seen as a mediator of a mediation system. It is used as an interface between the local schema of a database and the global schema defined by bio-ontologies. It is possible to detect all of the heterogeneous RDF views that are annotated with the same ontological term and then retrieve data from corresponding relational databases.

The RDF view generated by D2RQ contains the elements of the database schema: entities, attributes, keys (primary, foreign) and metadata, such as the database driver and host. The data contained in the relational databases are not included in the RDF view. Instances are retrieved directly from the databases. D2RQ API uses metadata from the RDF views to connect to the databases and to retrieve instances from them. The RDF view is queried with a SPARQL query; then, the D2RQ API transforms this query into an equivalent SQL query. Thus, there is no problem with keeping data up-to-date because the data are not physically exported.

**Figure 4:**
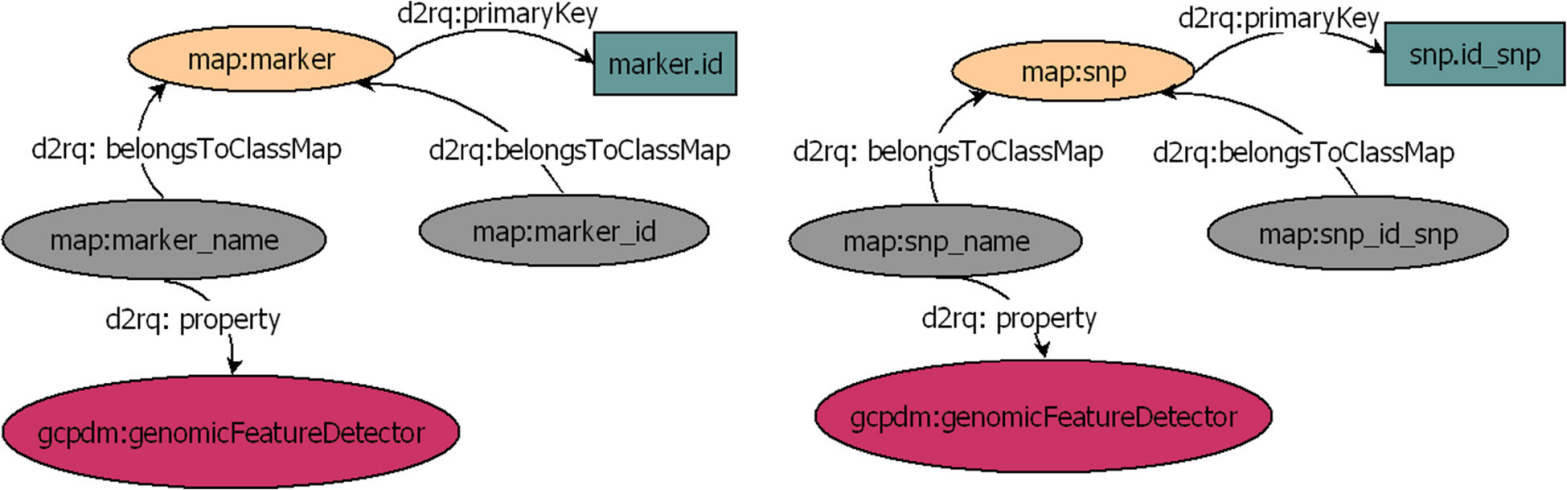
**Graph-based representation of annotated RDF views.** Each graph is the RDF representation of some part of a relational database. The *d2rq:belongsToClassMap* property links a column to a table. The *d2rq:primaryKey* property defines the primary key of a table. The *d2rq:property* property links a node to a semantic annotation. The columns *marker_name*, from the table *marker*, and *snp_name*, from the table *snp*, are both annotated with the same term: *genomicFeatureDetector* from the GCP domain model ontology [27].

In the RDF view, the database schema is represented by a graph. Each node corresponds to an entity or attribute in the database, and each edge defines a relationship between two nodes. In RDF format, namespaces are used to uniquely identify each node. Namespaces provide a prefix for each node name. For example, the *map:marker* node (Figure 4) indicates the “marker” concept from the “map” vocabulary used by D2RQ to uniquely identify one RDF view and to map relational elements to the RDF view.

#### Automatic semantic enrichment of the RDF view with BioSemantic

The BioSemantic API automatically detects specific information related to the relational database schema and translates it into new properties that can be integrated into the RDF view. These metadata are then used for SPARQL query generation. This step can be seen as a semantic enrichment of the RDF view.

1. *Association tables*

For this purpose, we have developed an algorithm that detects association tables.

2. *Arity*

**Figure 5:**
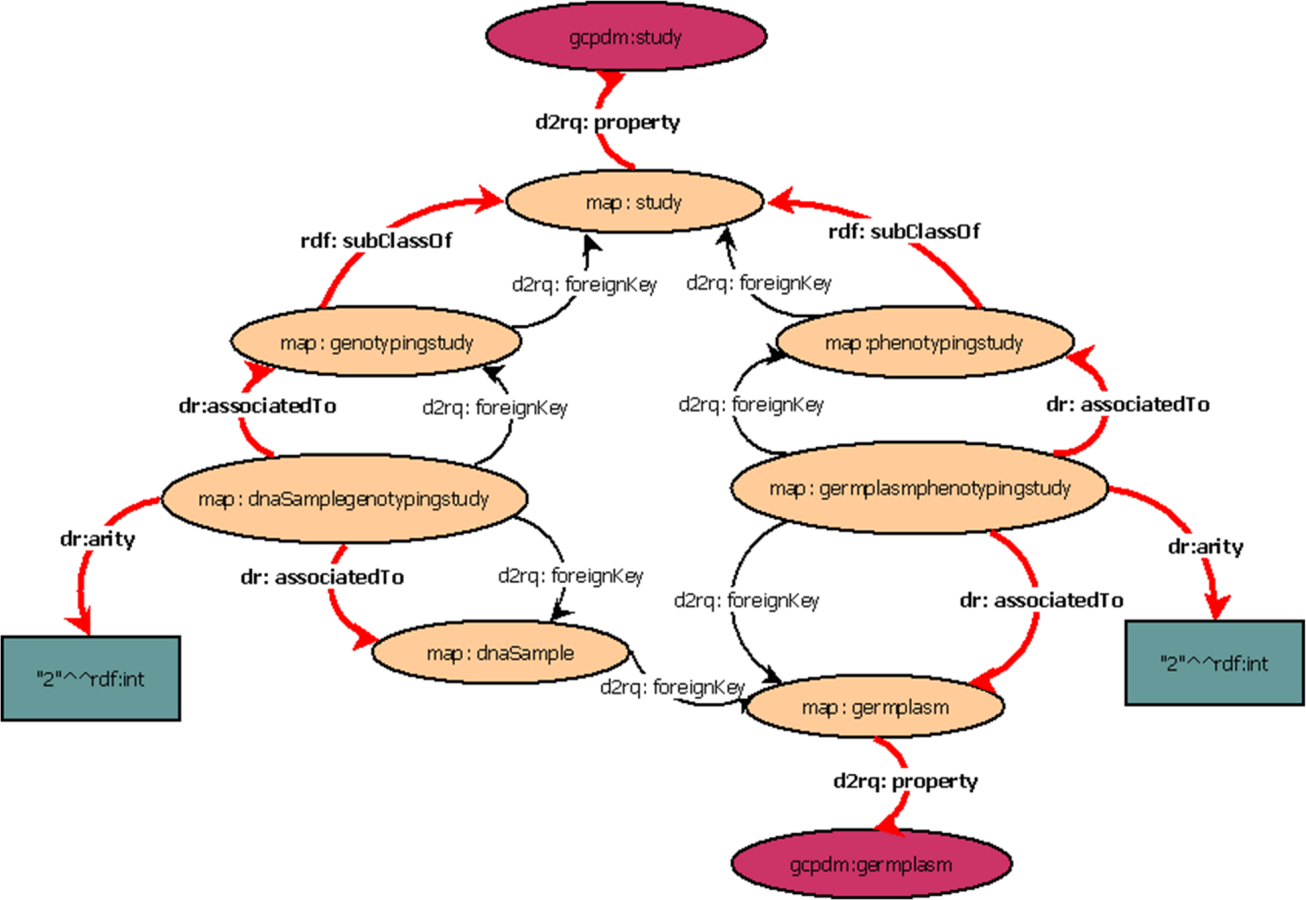
**Classification of the database table relationships.** Each light node represents a table of the relational database. Here, we only show the tables, and the columns are not represented. The dark nodes represent the semantic annotations. Each edge represents a property that is shared between 2 nodes. The new properties added by our method, *dr:associatedTo*, *dr:arity* and *rdf:subClassOf*, are indicated in bold.

We can also detect the arity of association tables, i.e., the number of foreign keys that they possess. The algorithm labels association tables in the RDF view with the *dr:associatedTo* property and indicates the arity with the *dr:arity* property (Figure 5).

3. *Inheritance, aggregation and composition*

There are many ways to transform inheritance relationships from an object-oriented conceptual model to a relational model [28]. For our algorithm, we detect relationships that result from the transformation of each class in an inheritance hierarchy into a table. We also detect tables that result from aggregation or composition relationships by using the identifying algorithm from [29]. We label these relationships in the RDF view with the *rdf:subClassOf* property (Figure 5).

#### Manual annotation with bio-ontological terms

The D2RQ language allows elements of the mapping file to be annotated with bio-ontological terms, which can be interpreted as semantic flags. Such flags can be used directly to query the relational database without any prior knowledge of the database schema or can be used to locate corresponding elements across databases (Figure 4). The annotation of the RDF view is performed manually by adding triples to the RDF view using a text editor and must be conducted by an expert familiar with both the database and the bio-ontology. In the plant biology domain, some ontologies are implemented in OBO format and do not provide URLs, in contrast to OWL ontologies. For this reason, the terms used to annotate the RDF view can be explained as URIs that do not resolve. Nevertheless, according to W3C standard it is recommended to use URLs that resolve.

### Automatic generation of the Semantic Web Service

Semantic annotations are used to select the inputs and outputs of a query. We can find a path in one RDF view by linking the inputs to the outputs. If such a path is found in the RDF view, then it is used to create a SPARQL query. To automate the creation of SPARQL queries, we implement an algorithm that is a single-pair variant of the shortest-path algorithm. Given an input graph, a source node and a destination node, the algorithm returns a path linking the two nodes through the graph. We add conditions to our shortest-path algorithm according to the types of relationships between the nodes, which can be either of the following: *(i)* relationships that correspond to association tables; or *(ii)* relationships that result from inheritance, aggregation, or composition in an object-oriented conceptual model. These conditions correspond to the metadata that is added to the RDF view during the automatic semantic enrichment step that is taken by the BioSemantic API.

#### Shortest-path algorithm with conditions

We parse the RDF view as though it were a graph, to find the shortest path linking two bio-ontological terms. These terms correspond to those selected as input and output for our WS.

We use a shortest-path detection approach based on the Dijkstra algorithm [30]. We add conditions to the weight path costs according to the properties classified in the previous step. In the weighting, we favour paths that correspond to binary associations. For the shortest paths that correspond to the *rdf:subClassOf* property (inheritance, aggregation or composition), we aggregate the different paths found. For example, in Figure 5, the *rdf:subClassOf* property allows a study to be considered a *genotypingStudy* or a *phenotypingStudy*. The data recorded in these two tables are complementary and are non-redundant. Indeed, the path linking *gcpdm:study* to *gcpdm:germplasm* is the combination of both paths:

***Path 1:****map:study- > map:genotypingstudy- > map:dnasamplegenotypingstudy*

- > map:dnasample- > map:germplasm

***Path 2:****map:study- > map:phenotypingstudy- > map:germplasmphenotypingstudy*

- > map:germplasm

These paths are not stored; instead, they are dynamically detected and are used to create a SPARQL query.

#### Generation of SPARQL queries

The detected path contains all of the information that is required for the automatic creation of a SPARQL query. For a given set of input/output bio-ontological terms and a given RDF view, only one SPARQL query can be created. The query below corresponds to the link between *gcpdm:study* and *gcpdm:germplasm*. *SELECT DISTINCT ?study_name ?germplasm_name WHERE {*

?study_id gcpdm:study ?study_name.

FILTER regex(?study_name,“^name_of_the_study$”).

{

?genotypestudy_id vocab:genotypingstudy_id_study ?study_id.

?key vocab:dnasamplegenotypingstudy_id_genotypingstudy ?genotypestudy_id.

?key vocab:dnasamplegenotypingstudy_id_dnasample ?dnasample.

*?dnasample vocab:dnasample_id_germplasm ?germplasm_id.*     ***Path 1***

?germplasm_id gcpdm:germplasm ?germplasm_name.

}

UNION {

?phenotypestudy_id vocab:phenotypingstudy_id_study ?study_id.

?key vocab:germplasmphenotypingstudy_id_phenotypingstudy ?phenotypestudy_id.

*?key vocab:germplasmphenotypingstudy_id_germplasm ?germplasm_id.*  ***Path 2***

?germplasm_id gcpdm:germplasm ?germplasm_name.

}

}

The first line of the query defines the attributes that correspond to the input and output of the WS. The third line is always a FILTER condition. This filter applies to the input attribute, which can be a literal or a regular expression. In our example, it is possible to retrieve the names of the germplasms that are used in a study by using names that begin with A and the regular expression “A.*”.

#### Automatic creation of the Semantic Web Service

**Figure 6:**
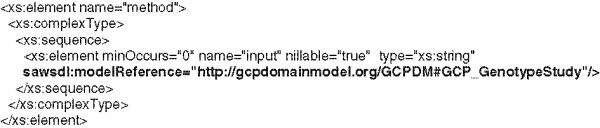
**SAWSDL annotation.** The semantic annotation is represented in bold and tags the input of our Semantic Web Service with the GCP_GenotypeStudy term from the GCP domain model ontology.

The SPARQL query is automatically integrated into a WS template. The WS is annotated with the bio-ontological terms previously selected as input and output for the query. According to the recommendations of the EMBRACE project [31] and the W3C, we use the Semantic Annotations for WSDL (SAWSDL) [32] to add semantic annotations to the WSDL (Web Services Description Language) components. The use of SAWSDL offers three main advantages: *(i)* it is compatible with the WSDL standard; *(ii)* it is lighter than other computing standards (i.e., WSMO (Web Service Modeling Ontology) and WSDL-S (Web Service Semantics)); and *(iii)* it is recommended by the W3C. Indeed, the input/output of our SWS are annotated using the *sawsdl:modelReference* attribute, specifying the association between an WSDL component and a bio-ontological term (Figure 6).

One SWS is created for each detected SPARQL query. All of the SWS annotated with the same input/output concepts can be easily detected and used for data integration. After the SWS is created, it can be registered in Web Service registries, such as BioCatalogue [33].

## Implementation

Our method is implemented in Java. The RDF views are created using the d2rq 0.7 library, and the RDF files are parsed using the Jena 2.5.7 library. The SWS are automatically deployed on a Tomcat 6.0 server using Axis2.

## Results

### Use case

We have created a use case integrating *Oryza sativa* (rice) data from distributed relational databases: Gramene [9], TropGene [34] and Ensembl [35]. Both the Gramene and TropGene databases have QTL data associated with traits, and these traits can be associated with concepts from the Trait Ontology. We wanted to compare the rice QTLs from the two resources, Gramene and TropGene, and to extract related genomic annotations from the Ensembl rice module.

We first used BioSemantic to create the SWS. We then used Taverna [4] to create a workflow by connecting BioSemantic SWS with external public WS. In this manner, we could verify the compatibility of BioSemantic SWS with standard WSDL WS. To increase the speed of querying over huge tables, we used a local copy of the Markers tables of Gramene; however, our example performed adequately using a remote access to the Gramene public database.

**Figure 7:**
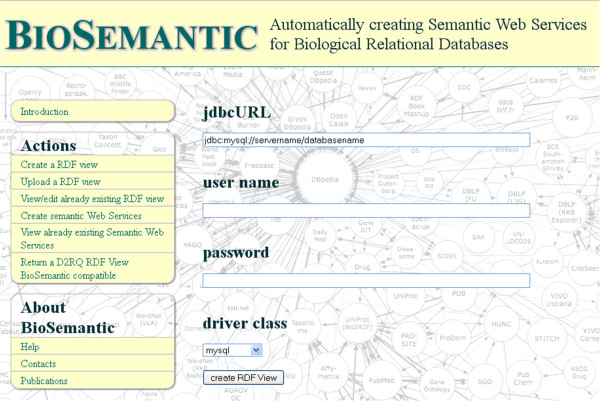
**BioSemantic form for automatic D2RQ RDF view creation.** For RDF view creation, the user must fill in all fields of the form. The left menu, known as “Actions”, contains all available BioSemantic actions.

All automatic steps can be performed directly on the BioSemantic Web user interface (Figure 7).

#### Steps involving SWS creation and using the BioSemantic Web user interface

A simple form must be completed to configure database access and to automatically create RDF views for the TropGene and Gramene databases (Figure 7). The RDF views can then be downloaded to perform semantic annotations. In our example, we annotated RDF views using one concept from the EDAM ontology [31]. The elements of the RDF views were annotated with the same ontological concept, known as edam:1093. For readability, we choose to represent this concept by its name edam:sequence_accession in our example. This annotation is added to triples corresponding to the `marker`.`name` column of the RDF View of TropGene. The annotation is represented below in bold type.

map:marker_name a d2rq:PropertyBridge;

d2rq:column "marker.name";

*d2rq:property edam:sequence_accession;*

d2rq:belongsToClassMap map:marker;

An annotation with the same term is added to triples corresponding to the `marker`.marker_acc` column of Gramene. The annotation is represented below in bold type.

map:marker_marker_acc a d2rq:PropertyBridge;

d2rq:column "marker.marker_acc";

*d2rq:property edam:sequence_accession;*

d2rq:belongsToClassMap map:marker;

The same ontological term is then used to annotate different database schemas. The BioSemantic Web interface allows users to upload the annotated RDF views to visualise the list of available RDF views in the repository, to download one of the views in order to view/add/modify annotations and to visualise the list of ontology and concept terms currently used into the RDF repository. This interface also allows users to automatically add BioSemantic annotations to a pre-existing D2RQ RDF view. Some projects use D2RQ, which means that some RDF views are currently annotated with domain ontologies. This functionality allows users to return these RDF views to BioSemantic compatibility without manual steps.

**Figure 8:**
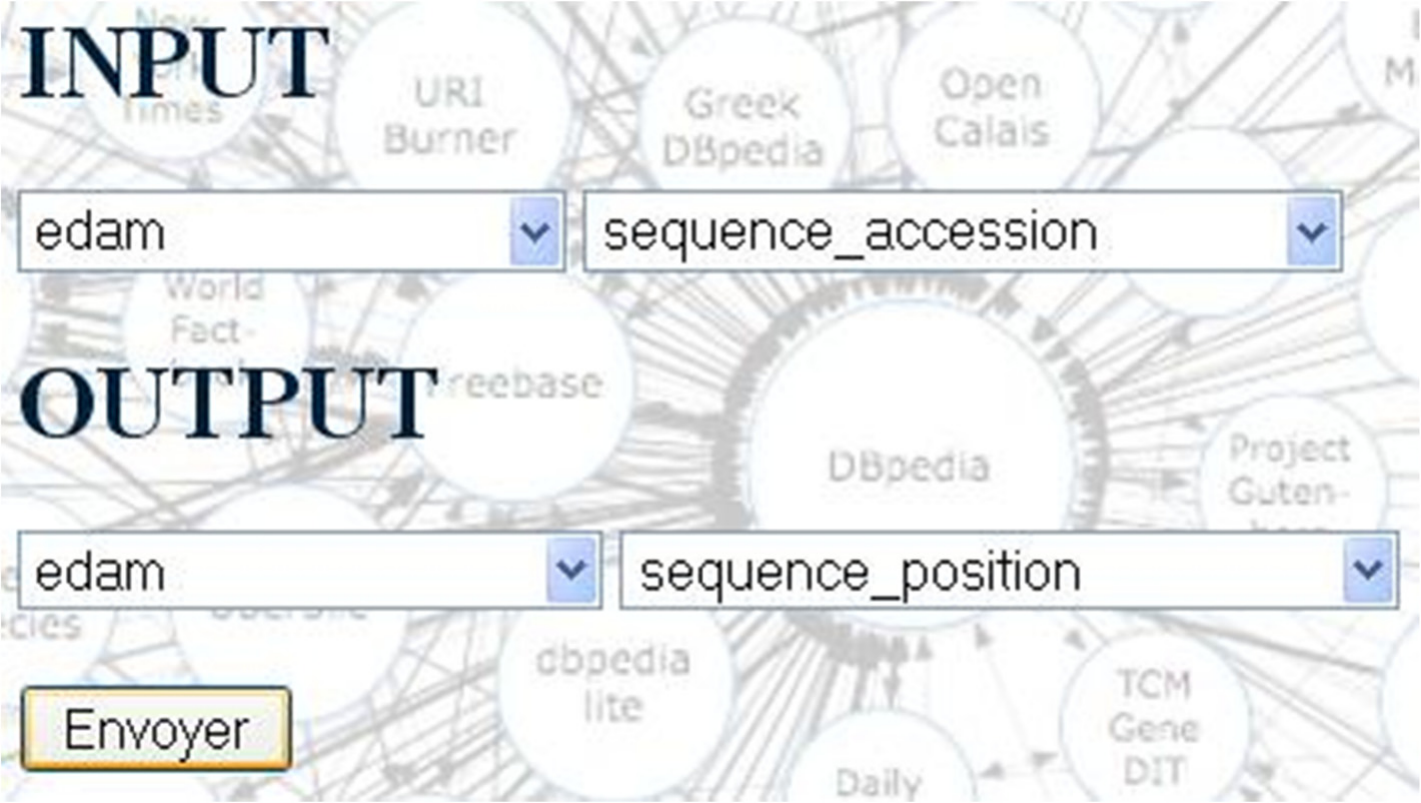
**BioSemantic form for input/output concept selection.** These concepts will be used to detect a path and to annotate the input/output of the SWS. The user can only select the prefix and concepts used to annotate a previously registered RDF view.

**Figure 9:**
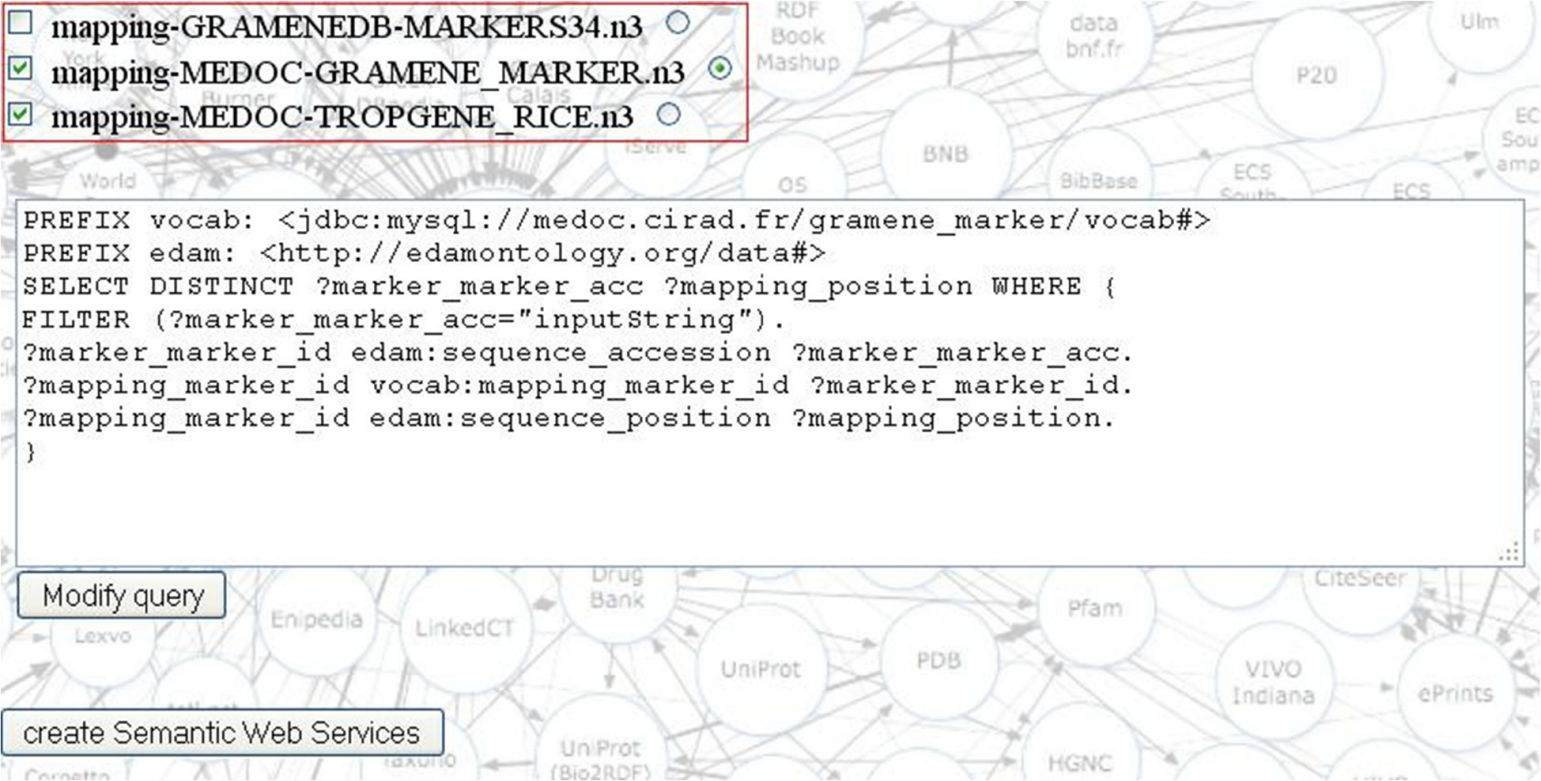
**BioSemantic form for RDF view selection and query visualisation/edition.** The red rectangle contains the name of the RDF views annotated with both input/output concepts. The checkbox before each name allows for the selection of a view for SWS creation. The radio button after the name of an RDF view allows for query visualisation/edition. When all desired RDF views are selected, a simple click creates the SWS.

After selection of the input/output bio-ontological terms (Figure 8), the BioSemantic application displays the list of RDF views containing these annotations (the red box in Figure 9). The checkbox before the name of an RDF view allows the user to select the SWS that he would like to create. By clicking on the radio button, the corresponding SPARQL query is displayed. It is then possible to validate the automatically generated query or to modify it (e.g., add more filters). A simple click on a button then creates SWS files and deploys them.

#### Workflow creation

BioSemantic SWS can be obtained directly with their WSDL localisation. In this use case, we chose to compose SWS as a workflow in Taverna. Taverna makes it possible to easily create a workflow, to visualise the progress of the running workflow and to save the workflow for the purpose of sharing it.

**Figure 10:**
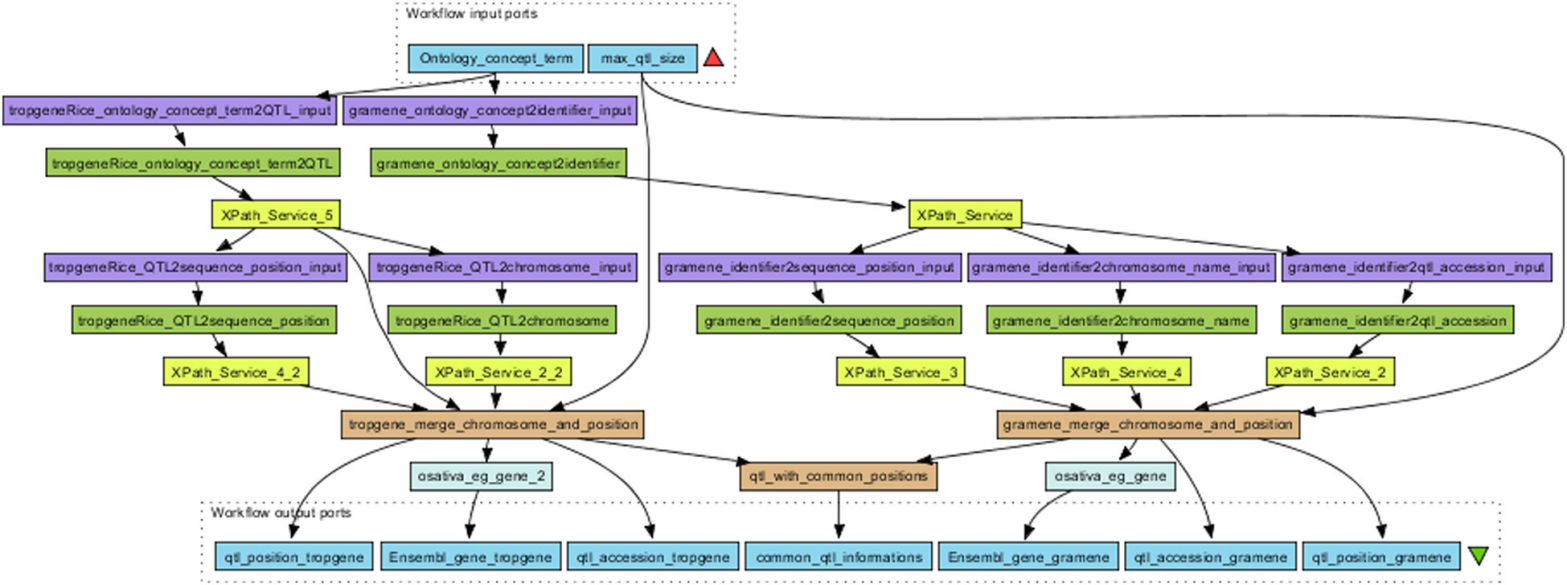
**Use case workflow created with Taverna 2.** The workflow contains BioSemantic SWS querying for both the TropGene and Gramene databases for QTL information retrieval and also contains BioMart WS querying EnSembl for gene information retrieval. This workflow also detects QTLs from TropGene and Gramene when both are annotated with the same TO term and have the same mapping position.

Our workflow (Figure 10) contains 7 BioSemantic SWS (green boxes). Yellow and purple boxes correspond to bricks that transform the inputs/outputs of the SWS and then allow for composition. For a given trait and QTL maximum size, BioSemantic SWS retrieve the Gramene and TropGene accession numbers of the QTLs along with their mapping positions in *Oryza sativa*. We have created a Beanshell Taverna brick (orange box) to retrieve the Gramene and TropGene QTLs that are mapped in the same genomic region. Two other bricks allow for the compatibility of the BioSemantic SWS with the Ensembl BioMart WSs. Indeed, we added Ensembl BioMart WS (blue boxes) to retrieve genes that are present in the mapping genomic interval of a given QTL. The yellow and purple boxes are shims that are added in Taverna. The purples boxes allow Taverna to manipulate BioSemantic SWS input. They are created automatically by Taverna. The yellow boxes are XPath expressions that allow Taverna to be compatible with BioSemantic SWS output. All of the yellow boxes are identical, and their creation is fast. However, the presence of these shims does not allow automation of BioSemantic SWS compositions.

In brief, our workflow retrieves the following rice information from TropGene, Gramene and Ensembl:

Accession number of the QTLs associated with a given trait,

Pair-based position of the mapping of these QTLs,

All of the genes in the mapping interval of a given QTL, and

QTLs with a common mapping position between TropGene and Gramene.

This workflow can be downloaded in my Experiment [36].

### Available Semantic Web Services

**Figure 11:**
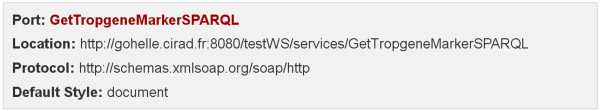
General information about the GetTropgeneMarkerSPARQL Web Service registered in the BioCatalogue.

**Figure 12:**
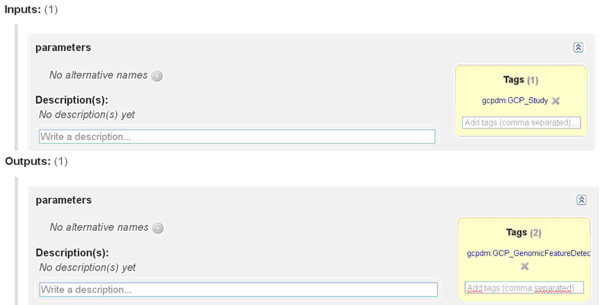
**BioCatalogue input/output annotations for the GetTropgeneMarkerSPARQL semantic Web Service.** Our bio-ontological terms correspond to the input/output tags in the BioCatalogue.

We developed other SWS for our own databases, including TropGene and OryGenesDB, a database of functional rice genomics data [37], as well as from external databases such as Gramene and SINGER [38], a multi-crop germplasm database. We annotated the database schemas with concepts from the Crop Ontology [39], the GCP Domain Model [15], the Sequence Ontology [40] and the EDAM ontology [31]. Some of these generated WS are available in the BioCatalogue (Figures 11 and 12).

### Benchmarks

With regard to automatically generated SPARQL queries, we are aware that, in some cases, there are multiple possible paths, each of which can be semantically valid depending on the query semantics. Our system identifies the “best” shortest-path with conditions favouring binary table associations and combines the paths corresponding to inheritance, aggregation and composition. However, a manual validation test for the automatically generated SWS is still recommended. Indeed, the SWS that we generated and tested were all validated by the database managers and/or users. Regardless of the validation ability, the main benefit of our platform is that it enables the rapid creation of new and easily detectable SWS.

#### SPARQL query generation

We have tested the speed of SPARQL query generation with two different biological databases: *(i)* TropGene, a relational database that contains 90 tables and 15 million records; and *(ii)* OryGenesDB, which contains 11 tables and 22 million records. Although SPARQL query generation is only performed during the first step of Web SWS generation and not during the SWS execution, we also measured the time required for this step. This time depends on the database schema but also strongly depends on the presence of inheritance relationships (Table 1). In this table, when inheritance relationships are present, we include the lengths of the paths to be aggregated. The creation of a query without inheritance relationships takes less than 2 seconds. However, creating a query using the same database schema with 4 inheritance relationships takes 15 seconds. In general, complex SPARQL queries can be created in a matter of seconds.

**Table 1:** Estimating the time required for SPARQL query creation

**Number of tables**	**Inheritance relationship**	**Length of the path**	**Time (seconds, ± 0.1)**
11	no	2 nodes	1.2
90	no	4 nodes	2.0
90	yes	4-3-2-6 nodes	14.6

#### SPARQL query execution

The time required for query execution varies significantly for different databases and strongly depends on the number of records to be retrieved. Table 2 compares the time required for SQL query execution and SPARQL query execution. We did not compare with the time required for the querying RDF dump of a relational database because some databases can contain more than 100 million tuples. The RDF dump will then contain more than 100 million triples, and triplestore query performances decrease with the number of triples. When the triplestore contains more than 100 million triples, SPARQL to SQL approaches are fastest [41]. The time required for SQL query execution was measured in Eclipse using the *java.sql* library. The time required for SPARQL query execution was measured using the AJAX-based SPARQL Explorer tool of the D2R Server.

**Table 2:** Comparison of the time required for SQL and SPARQL query execution

**Number of tables**	**Inheritance relationship**	**Length of the path**	**Number of results**	**SQL query (seconds, ± 0.1)**	**SPARQL query in D2R Server (seconds, ± 0.1)**
90	no	4	860	0.4	1.4
90	no	4	1456	0.4	1.4
90	no	2	2055	0.8	2.3
90	yes	4-3-2-6	8071	1.1	4.2
90	no	3	12302	2.3	4.8

The SPARQL approach takes approximately 3-4 times longer to access data than a direct SQL query, but users can still retrieve more than 5000 results in a few seconds. The time required to display the SPARQL results in the AJAX-based SPARQL Explorer accounts, in part, for the differences in performance. Most of the overhead, however, comes from the transformation of SPARQL queries into SQL queries, which is performed using the D2RQ engine.

#### Semantic Web Services execution

Table 3 compares the time required for SWS execution using manually created SQL WS and our automatically generated SPARQL SWS. These Web Services query the TropGene database. Although manually created WS are faster than our automatically created SWS, the difference is not dramatic enough to affect the usability of our SWS.

**Table 3:** Comparison of the time required for Web Service execution using the SQL Web Services and automatically generated using the SPARQL Web Services

**Query**	**Number of results**	**SQL Web Services (seconds, ± 0.1)**	**SPARQL Web Services (seconds, ± 0.1)**
retrieves genotyping studies	7	0.2	1.0
retrieves germplasms for selected studies	860	0.4	1.0
retrieves markers for selected studies	1456	0.4	1.0

#### Validation of the SPARQL query results

We compared the data retrieval resulting from the three approaches (i.e., the Dijkstra algorithm, BioSemantic and a human SQL query builder) (Table 4). We refer to a human SQL query as a query that is manually written by an expert with good knowledge of the database schema. A first general observation demonstrates that the number of results is identical for BioSemantic queries and the manual SQL queries. BioSemantic globally retrieves more results than the Dijkstra algorithm. The gap for Query1 is explained because of the inheritance relationships missed by the Dijkstra algorithm. Indeed, in that case, BioSemantic detects these relationships and regroups the subdivided paths into the final query. Furthermore, BioSemantic preferentially selects binary association tables that promote more data retrieval. Both Query2 and Query3 correspond to a short path without inheritance but with several paths having the same node numbers. In that case, weighting the BioSemantic path favours binary associations, whereas the Dijkstra algorithm chooses the first detected path having a minimum node number. For Query2, BioSemantic favours the detection of a more pertinent path, whereas the same paths are detected for Query3. For Query4, no equivalent path guides to the same results; in other words, both algorithms select the same path. In each case, we manually verified that the retrieved data were identical.

**Table 4:** Comparing the number of retrieved data from the three approaches: Dijkstra algorithm, BioSemantic and human SQL query builder

	**Inheritance**	**Equivalent paths**	**Dijkstra**	**BioSemantic**	**Manual SQL**
Query 1	yes	no	1595	7212	7212
Query 2	no	yes	0	12302	12302
Query 3	no	yes	197	197	197
Query 4	no	no	2055	2055	2055

#### Comparison with other SWS platforms

We compared BioSemantic with other SWS platforms, such as BioMoby [20], SADI [19] and SSWAP [18] (Table 5). BioMoby adds semantic components to WSs by using an XML datatype ontology developed by WS developers. SSWAP is based on a five-class ontology allowing the definition of Web resources, inputs and outputs of the SWS, data structures and data providers. SADI is a set of fully standard-compliant SWS design patterns that simplify their publication. A SADI plugin has been developed. This plugin helps users to discover SADI SWS and to automatically compose them in workflows.

**Table 5:** Comparison with other SWS platforms

	**Semantic Web Standard**	**Annotations**	**WSDL compliant**	**Platform specific**	**Reasoner**	**Creation/ deployment**	**Query creation**
**BioMoby**	no	XML	yes	yes	no	semi-automatic	manual
**SSWAP**	yes	OWL	no	yes	yes	manual	manual
**SADI**	yes	OWL	yes	no	yes	semi-automatic	manual
**BioSemantic**	yes	SAWSDL	yes	no	no	automatic	automatic

In this comparison, we focused on the ability to create and use SWS because the other SWS approaches are not placed in the context of the automated creation of wrappers for relational databases.

We compared seven criteria: i) the exclusive use of SW standards; ii) the types of input and output annotation for SWS; iii) the compliance with SOAP/WSDL; iv) the constraint for clients to be platform specific; v) the ability of the platform to perform reasoning; vi) the degree of automation in the creation and deployment of SWS; and vii) the degree of automation of the query building.

All of the compared approaches use SW standards except for BioMoby, in which semantics come from the data type stored in an XML tree. In terms of output, SADI and SSWAP are based on OWL, and both developed their own SWS API to exploit OWL’s reasoning capabilities. BioSemantic uses the standard SAWSDL to semantically annotate the WSDL files.

BioMoby, SADI and BioSemantic are compliant with SOAP/WSDL protocols. Some of the approaches are platform specific (i.e., SSWAP and BioMoby), meaning that they require their own environment to process SWS. For example, SSWAP gains in speed and lightness but loses in genericity. BioMoby develops its own data type definition, allowing for an easy choreography of services, but requires clients to be compliant with the API. BioSemantic and SADI use standard clients to call their SWS.

In terms of reasoning abilities, SADI and SSWAP exploit OWL with semantic reasoners to highlight some relationships between classes. On the other hand, BioMoby exploits the taxonomic properties of XML to infer relationships between data types; however, BioMoby is less expressive than OWL. BioSemantic comes without reasoning capabilities. Initially, this task was to be performed by the SWS catalogue (i.e., BioCatalogue), but this function is not yet available.

The last two criteria define the degree of automation of these approaches. BioMoby and SADI allow for the creation and deployment of SWS skeletons without including core methods. BioSemantic is the only API that processes query creation. This automation is allowed by decoupling annotated RDF view creation and SWS creation. However, this automatic creation of SWS is still dependent on the manual RDF view annotation step performed by the data provider.

## Discussion

### Semantic limitations

#### OBO ontologies

The development of an ontology is a long community-based task in which participants decide on a consensus basis about term definitions and relationships between those terms. Currently, a large number of bio-ontologies exist and cover a large spectrum of biological domains.

Most of these ontologies are not developed in an OWL format; instead, they are in an OBO format, which follows the OBO Foundry principles [42], such as unique URI or formatted term/concept names.

Regarding the amount of work that is necessary to create an ontology, we decided to allow the annotation of RDF view using terms from OBO ontologies. However, that strategy could raise problems, such as the possible lack of a URL that could resolve these ontologies. However, even if OBO Foundry principles only recommend using unique URIs, a lot of already existing OBO ontologies are associated to URLs. Furthermore, if OBO ontologies do not use URLs that currently resolve, it is still possible to register them with online tools such as BioPortal or Ontology Lookup Service (OLS). In our case, we deployed an instance of OLS allowing publishing ontologies on the Web.

In our approach, the major limit from OBO ontologies comes from the low number of classes possessing restrictions along with the low number of different properties used (e.g., BioPortal notes that 8 properties are used in the GO, which possesses more than 38000 classes). Therefore, using those ontologies has a strong impact on BioSemantic by significantly limiting its semantic component.

#### Manual SWS composition

The SWS BioSemantic composition requires the development of shims. This requirement is a limit to the workflow creation that could be overtaken by creating a Taverna plugin or by making the BioSemantic framework compatible with SADI. Moreover, SADI already possesses a Taverna plugin. Furthermore, that compatibility could take advantage of a stronger semantic without being platform specific.

#### No use of existing framework

In BioSemantic, we choose to not reuse already existing frameworks such as BioMoby, SSWAP or SADI. Indeed, the purpose of these frameworks is to better organise semantic components, whereas the main purpose of BioSemantic is to separate the steps of publishing relational schema and the creation of SWS and then to automate the step of SWS creation.

During our work, we did not focus on making our approach compatible with an already existing framework. The main reason was that we did not want to be affected by the technical or compatibility limits of other WS or by the success of our approach depending on a specific framework. However, SSWAP and SADI are based on OWL, which allows the creation of SWS with stronger semantics than BioSemantic. Using BioSemantic in those frameworks could increase widely the semantic component of SWS created by BioSemantic and therefore automate their composition.

#### Differences between semantic and data type

The use of bio-ontology terms to annotate input/output allows for easier detection of our SWS by searching services with a standard vocabulary.

Annotations are composed of adding a semantic flag on a component of a database schema, which requires choosing which component of a schema will be annotated.

That step is performed manually, and does not guarantee that the same annotation will be associated with similar data. For example, we used the term gcpdm:study to annotate the name of a study because the only identifier of a study existing in the TropGene database is an auto increment with no scientific sense. If another curator uses the same term to annotate an identifier, the data returned by the two different services would not be comparable even if the two services return information on a genotyping study. That limit prevents the automatic composition of our services into workflows.

### Shortest path algorithm

#### One input and one output

The major limit of our query comes from the restriction to a single input concept and a single output concept. That restriction is because of the shortest path algorithm, which allows only the joining of a node of a graph to another node. That restriction implies that we create a query coming from a linear path in the graph that represents the database schema.

It would be interesting to modify our algorithm to find a path that links a number n of input nodes in our future query to n output nodes.

#### Retrieve one path

Currently, BioSemantic allows automatic query creations based on our shortest path algorithm. We plan to allow a user to choose between different paths. The visualisation of these paths, in which nodes correspond to database table names, will aid in user selection.

#### Self join detection

Furthermore, BioSemantic does not allow the creation of queries annotated with the same input and output concept as a consequence of using the Dijkstra algorithm. This functionality would be very interesting for orthologous or synonym detection for example.

We plan to implement a simple algorithm allowing the detection of all self joins that correspond with a given table. In fact, if a table has several self joins, then the path length found for each of them will be identical. For this reason, both the path visualisation in the graphical interface for the query creation and the algorithm of self join detection will overtake this limitation, allowing the user to select the name of the wanted association table, to link one table to itself and then to create the wanted query.

### Manual RDF view annotation

Future developments will concern the semantic annotation of RDF views, which is the only manual task in BioSemantic. This task could be a time-consuming task for database annotators if the database schema is large. Constraints for annotators arise many because they must be experts on database schemas and the ontology terms and must also manipulate RDF and D2RQ. We believe that this limitation could be partially overcome by creating a user interface for the annotation of RDF Views.

Our solution opens new perspectives for the development of SWS. However, we are still interested in adding more functionality, such as the automatic generation of links between database schemas and existing ontologies. We are currently exploring the use of automatic schema-matching tools developed in the context of the WebSmatch platform [43].

### Performance problems with FILTER

Input variables of our services can be regular expressions or literal expressions. Those variables are detected when WS is used and will lead to the use of a different SPARQL FILTER. A regular expression used in a WS input could raise query problems, for example, it could create memory errors when querying tables that contain hundreds of thousands of tuples.

## Conclusions

BioSemantic is a framework that is designed to speed the development of Semantic Web Services for existing relational biological databases. This framework has the specific capability of separating the publishing step of the relational schema from the SWS creation. Data consumers can then create Semantic Web Services without knowledge of the resource schema. Currently, it automatically creates and semi-automatically annotates RDF views that enable the automatic generation of SPARQL queries. These queries are created by the following steps: *(i)* the selection of input and output ontological terms using a Web interface that is available in the BioSemantic API; *(ii)* the automatic detection of a path linking inputs to outputs; and *(iii)* the use of the path to automatically generate a SPARQL query. Semantic Web Services are also automatically created and deployed.

## Availability and requirements

•**Project name:** BioSemantic

•**Project home page:**http://southgreen.cirad.fr/?q=content/Biosemantic

•**Operating system(s):** Platform independent

•**Programming language:** java

•**Restrictions on use by non-academics:** no limitations

## Competing interests

The authors declare that they have no competing interests.

## Authors’ contributions

JW developed and tested the Java code. All of the authors contributed to the design of the software architecture and the development of the appropriate methods. All of the authors read and approved the final version of the manuscript.
